# Genome-Wide Identification and Expression Analysis of the Cyclic Nucleotide-Gated Channel Gene Family in *Zoysia japonica* under Salt Stress

**DOI:** 10.3390/ijms251810114

**Published:** 2024-09-20

**Authors:** Shu-Tong Li, Wei-Yi Kong, Jing-Bo Chen, Dong-Li Hao, Hai-Lin Guo

**Affiliations:** The National Forestry and Grassland Administration Engineering Research Center for Germplasm Innovation and Utilization of Warm-Season Turfgrasses, Jiangsu Key Laboratory for the Research and Utilization of Plant Resources, Institute of Botany, Jiangsu Province and Chinese Academy of Sciences (Nanjing Botanical Garden Mem. Sun Yat-Sen), Nanjing 210014, China; lishutong@jib.ac.cn (S.-T.L.); kongweiyi@jib.ac.cn (W.-Y.K.); chenjingbo@jib.ac.cn (J.-B.C.)

**Keywords:** *Zoysia japonica*, *CNGC*, salt stress, genome-wide analysis, qRT–PCR

## Abstract

Salt stress severely inhibits plant growth. Understanding the mechanism of plant salt tolerance is highly important to improving plant salt tolerance. Previous studies have shown that nonselective cyclic nucleotide-gated ion channels (*CNGCs*) play an important role in plant salt tolerance. However, current research on *CNGCs* mainly focuses on *CNGCs* in glycophytic plants, and research on *CNGCs* in halophytes that exhibit special salt tolerance strategies is still scarce. This study used the halophilic plant *Zoysia japonica*, an excellent warm-season turfgrass, as the experimental material. Through bioinformatics analysis, 18 members of the *CNGC* family were identified in *Zoysia japonica*; they were designated *ZjCNGC1* through *ZjCNGC18* according to their scaffold-level chromosomal positions. *ZjCNGCs* are divided into four groups (I–IV), with the same groups having differentiated protein-conserved domains and gene structures. *ZjCNGCs* are unevenly distributed on 16 scaffold-level chromosomes. Compared with other species, the *ZjCNGCs* in Group III exhibit obvious gene expansion, mainly due to duplication of gene segments. The collinearity between *ZjCNGCs*, *OsCNGCs*, and *SjCNGCs* suggests that *CNGCs* are evolutionarily conserved among gramineous plants. However, the Group III *ZjCNGCs* are only partially collinear with *OsCNGCs* and *SjCNGCs*, implying that the expansion of Group III *ZjCNGC* genes may have been an independent event occurring in *Zoysia japonica*. Protein interaction prediction revealed that ZjCNGCs, calcium-dependent protein kinase, H^+^-ATPase, outwardly rectifying potassium channel protein, and polyubiquitin 3 interact with ZjCNGCs. Multiple stress response regulatory elements, including those involved in salt stress, are present on the *ZjCNGC* promoter. The qPCR results revealed differences in the expression patterns of *ZjCNGCs* in different parts of the plant. Under salt stress conditions, the expression of *ZjCNGCs* was significantly upregulated in roots and leaves, with *ZjCNGC8* and *ZjCNGC13* showing the greatest increase in expression in the roots. These results collectively suggest that *ZjCNGCs* play an important role in salt tolerance and that their expansion into Group III may be a special mechanism underlying the salt tolerance of *Zoysia japonica*.

## 1. Introduction

Salt stress is an extreme environmental stress condition that can cause excessive accumulation of sodium ions in plants, resulting in toxicity to plant cells and severe inhibition of plant growth [1]. More than 20% of irrigated land worldwide is reported to be affected by salt stress [2]. Under salt stress conditions, the net accumulation of Na^+^ in plants is determined mainly by the rates of passive unidirectional inflow and active expulsion of Na^+^ [3]. Imbalances in intracellular sodium ion homeostasis are detrimental to many physiological and biochemical processes in plant cells. To survive in salt stress environments, plants use three main strategies: (1) limiting the flow of Na^+^ into the plant through the roots and expelling Na^+^ from the roots; (2) distributing Na^+^ to less sensitive tissues such as older leaves and isolating excess Na^+^ in vacuoles; and (3) secretion of Na^+^ via salt glands [4,5]. However, the molecular mechanisms underlying the above physiological processes remain less clear [6,7]. Therefore, analysis of the salt tolerance mechanisms of plants, especially halophilic plants, at the molecular level is highly important to improving plant salt tolerance.

Cyclic nucleotide-gated ion channels (CNGCs) are tetrameric cation channels that mediate the uptake of cations such as Na^+^, K^+^, and Ca^2+^. CNGCs are activated by cyclic nucleotide monophosphates (cNMPs), including 3′,5′-cyclic adenosine monophosphate (cAMP) and 3′,5′-cyclic guanosine monophosphate (cGMP) [8,9,10,11,12,13,14,15]. CNGCs are not only essential for plant nutrition but are also involved in plant development, abiotic stress, and immunity through calcium (Ca^2+^) signaling [16,17,18,19,20,21]. The *CNGC* gene was first discovered in animals, and Fesenko et al. reported that cGMP can directly activate light-dependent channels, namely, cyclic nucleotide-gated ion channels (CNGCs), in retinal rods, thus opening the prelude to CNGC research [22]. The CNGC ion channels in plants were first discovered in 1998 during screening of the *Hordeum vulgare* CaM-binding transporter (HvCBT) in barley [23]. Numerous species of CNGCs have since been studied. Previous studies have shown that the plant CNGC family can be divided into four main groups (Groups I–IV); Group IV is further divided into two subgroups, IV-a and IV-b [24]. There are 20 members of the *CNGC* gene family in *Arabidopsis thaliana* [25], 16 members in rice [26], and 16 members in foxtail millet [27]. Yeast mutants expressing *AtCNGC3*, a member of Group I, are more sensitive to high salt concentrations and accumulate more Na^+^ than cells transfected with an empty vector, and cngc3 transgenic seedlings are more resistant to salt toxicity than wild-type seedlings [28]. Knockout of *AtCNGC10*, a member of Group I, enhances salt tolerance in Arabidopsis, whereas its overexpression reduces salt tolerance, indicating that *AtCNGC* negatively regulates salt tolerance in Arabidopsis [29]. *AtCNGC5*, a member of Group II, and *AtCNGC17*, a member of Group III, also confer salt tolerance [19]. These results indicate that *CNGCs* play an important role in plant salt tolerance.

The activity of CNGCs is regulated not only at the transcriptional level but also at the protein level [30]. The genes encoding *AtCNGC19* and *AtCNGC20* are differentially expressed in Arabidopsis roots and aboveground parts in response to salt stress. *AtCNGC19* is expressed mainly in roots during early growth, whereas *AtCNGC20* shows a linear increase in expression in the mesophyll cells surrounding leaf veins, with the highest expression observed in mature and senescent leaves. After infection with sheath blight, expression of the genes encoding *LeCNGC17* and *LeCNGC18* was downregulated, and silencing of these two genes increased tomato resistance to *F. oxysporum*. Similarly, the overall expression levels of *BnCNC27*, *48*, and *9* were downregulated after infection by *F. oxysporum* [31,32]. *BoCNGC17* expression in cabbage was significantly upregulated under conditions of low-temperature stress [33]. At the protein level, the coordination of soybean GsCNGC20-d and GsCDPK29 is involved in the adaptive response of salt-tolerant soybeans to salt stress [34]. AtCNGC2 interacts with AtCNGC4 and AtCaM and plays important roles in plant immunity, development, and ion homeostasis [35,36]. Plasma membrane-localized AtCNGC12 and AtCNGC14 interact with CaM by binding Ca^2+^ to the IQ domain of the AtCNGC protein, thereby activating AtCNGC channels and playing important roles in immunity and tropism [37,38]. Moreover, AtCNGC17, AtAHAs, and the receptor AtBAK1 may form a functional core unit that regulates plant growth by coordinating proton efflux and cation uptake through the plasma membrane [39]. Taken together, the above studies suggest that CNGC activity is tightly regulated at the transcriptional and protein levels in response to various stress stimuli, including salt stress.

Members of the *CNGC* gene family have been identified and preliminarily studied in glycophytic plants such as Arabidopsis [25], rice [26], foxtail millet [27], tomato [32], wheat [40], Chinese cable [41], and tobacco [42]. However, the identification of *CNGC* family members in halophytes is still rare. *Zoysia japonica* is considered an excellent perennial warm-season turf that exhibits good drought and salt tolerance. *Zoysia japonica*, *Zoysia matrella*, and *Zoysia macrostachya* are halophytes [43,44,45] with greater salt tolerance than most other plants. Understanding the mechanism of salt tolerance in halophytes may provide new ideas for increasing plant salt tolerance. Since the 1990s, studies of the salt tolerance of the halophyte *Zoysia japonica* have attracted increased interest. Although many studies of the molecular mechanism of salt tolerance in *Zoysia japonica* have been conducted [46,47,48,49], there is little information on the *ZjCNGC* gene of *Zoysia japonica*. Therefore, we conducted a genome-wide identification of the *ZjCNGC* gene family in *Zoysia japonica*. A comprehensive analysis of the identified *CNGC* genes, including protein sequence characteristics, structural domains, motifs, phylogenetic relationships, gene structure, collinearity analysis, and cis regulatory elements, was conducted. Furthermore, quantitative PCR (qPCR) was used to analyze the patterns of expression of the *CNGC* gene in different tissues and under salt treatment. It is expected that the above research will reveal information about the members of the *CNGC* family in *Zoysia japonica*, laying the foundation for the subsequent use of this gene family to improve plant salt tolerance. All 18 *ZjCNGCs* were upregulated under salt stress, suggesting that they play important roles in the salt tolerance of *Zoysia japonica*. Compared with other species, *ZjCNGC* has undergone gene expansion in Group III; this may be a special mechanism for achieving salt tolerance in the halophyte *Zoysia japonica*.

## 2. Results

### 2.1. Identification and Physicochemical Properties of CNGC Genes in Zoysia japonica

To accurately identify the *ZjCNGC* gene family members of *Zoysia japonica* at the whole-genome level, the annotated protein sequences from the genome of *Zoysia japonica*, the genomes of the related species millet and rice, and the genome of Arabidopsis were screened for proteins containing conserved ion transport (PF00520.30) or cNMP-binding (PF00027.28) domains. The selected candidate protein sequences were aligned with the UniProt database via BLASTP to determine the target protein annotated as a cyclic nucleotide-gated ion channel (CNGC). A total of 20 ZjCNGC proteins, 16 SiCNGC proteins, 16 OsCNGC proteins, and 20 AtCNGC proteins were preliminarily obtained. However, after amino acid sequence alignment of the CNGC homologous proteins, only three ZjCNGC proteins (Zjn_stc00031.g02390.1sm.mk, Zjn_stc00135.1.g00520.1.am.mkhc, and Zjn_stc00181.g00240.1.sm.mk) were found to have complete sequences. To improve the integrity of the identification of the remaining ZjCNGC proteins, we further searched the full-length transcriptome of *Zoysia japonica* using the same method. By referencing the Nagirizaki genome for gene redundancy removal, we obtained a total of 11 ZjCNGC-Iso proteins (PB. 1.1 through PB. 11.1). The *ZjCNGC* gene was then reannotated using the *ZjCNGC-ISO*, *OsCNGC*, *SiCNGC*, and *AtCNGC* gene sequences, and 21 ZjCNGC proteins were identified via manual correction of the sequences. The sequences of three of the proteins (Zjn_sc00017.1.g00010.1.am.mk, Zjn_sc00351.1.g00010.1.am.mk and Zjn_sc04840.1.g00010.1.am.mk) were truncated due to gaps in the genomic assembly and could not be included in the subsequent analysis. Finally, 18 *ZjCNGC* genes with complete sequences were identified; they were designated *ZjCNGC1* through *ZjCNGC18* on the basis of the scaffold from small to large (Table 1).

The physicochemical properties of the 18 ZjCNGC proteins differ greatly. The number of amino acid residues ranges from 179 to 766, and the molecular weights (MWs) of the proteins range from 20.52 to 86.95 kDa. All 18 are basic proteins (isoelectric point (pI) greater than 8.00). The mean hydrophilicity (GRAVY) of the 18 proteins is negative, indicating that all except ZjCNGC5 and ZjCNGC9, whose GRAVY values are 0.09 and 0.05, respectively, are hydrophilic proteins. Most of the ZjCNGC members have instability indices (IIs) greater than 40, indicating that they are unstable proteins. ZjCNGC8 and ZjCNGC11, however, are stable proteins, with IIs of 38.09 and 38.34, respectively. The results of subcellular localization prediction revealed that most of the CNGC proteins are located in chloroplasts, a few are located in the plasma membrane, and only ZjCNGC15 is located in the nucleus. The results of transmembrane domain analysis revealed that the number of transmembrane regions in the ZjCNGCs ranges from 0 to 7. ZjCNGC11 does not have a transmembrane region, and ZjCNGC6 has the highest number of transmembrane regions (Table 1). The significant differences in the basic properties of ZjCNGC family member proteins suggest that they may have different functions.

### 2.2. Protein Phylogenetic Tree

**Figure 1:**
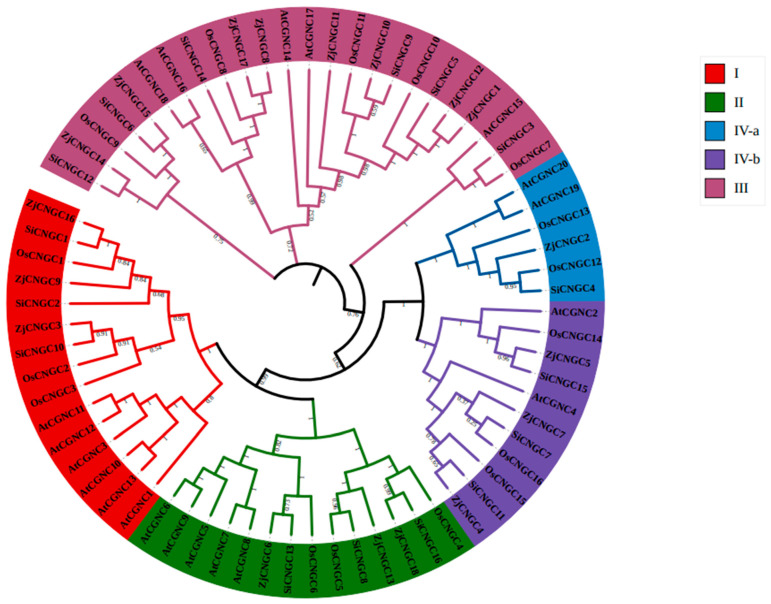
Construction of a protein tree of AtCNGCs, OsCNGCs, SiCNGCs, and ZjCNGCs via the maximum likelihood method with 1000 bootstrap values. Different colors indicate specific CNGC groups; Groups I, II, III, IVa, and IVb are shown in red, green, blue, purple, and light purple, respectively.

To further understand the evolutionary relationships among the *CNGC* family genes in *Zoysia japonica*, MEGA 7 was used to construct a phylogenetic tree of the CNGC protein sequences (70 members) from *Zoysia japonica* (18), *Arabidopsis thaliana* (20), *Oryza sativa* L. (16), and *Setaria italica* (16) (Figure 1). The results indicated that the CNGC family members can be divided into four groups (I–IV), with Group IV further divided into IV-a and IV-b. *Zoysia japonica* has three ZjCNGCs (ZjCNGC3, 9, and 16) in Group I, three (ZjCNGC6, 13, and 18) in Group II, eight (ZjCNGC1, 8, 10, 11, 12, 14, 15, and 17) in Group III, and four (ZjCNGC2, 4, 5, and 7) in Group IV. *Arabidopsis thaliana* has six AtCNGCs (AtCNGC1, 3, 10, 11, 12, and 13) in Group I, five (AtCNGC5, 6, 7, 8, and 9) in Group II, five (AtCNGC14, 15, 16, 17, and 18) in Group III, and four (AtCNGC2, 4, 19, and 20) in Group IV. Rice has three OsCNGCs (OsCNGC1, 2, and 3) in Group I, three (OsCNGC4, 5, and 6) in Group II, five (OsCNGC7, 8, 9, 10, and 11) in Group III, and five (OsCNGC12, 13, 14, 15, and 16) in Group IV. Foxtail millet has three SiCNGCs (SiCNGC1, 2, and 10) in Group I, three (SiCNGC8, 13, and 16) in Group II, six (SiCNGC3, 5, 6, 9, 12, and 14) in Group III, and four (4, 7, 11, and 15) in Group IV. The above results indicate that although the number of members of the CNGC family present in *Zoysia japonica* is moderate among the four compared species, *Zoysia japonica* has more Group III members than do the other three species.

### 2.3. Analysis of Gene Structure and Conserved Protein Structure

**Figure 2:**
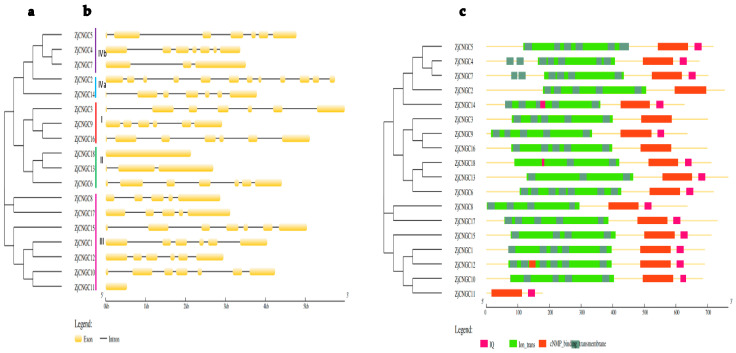
Schematic representation of the protein tree, gene structure and domain analysis of 18 ZjCNGCs. (**a**) Construction of a protein tree of 18 ZjCNGCs via the maximum likelihood method using the protein sequences of ZjCNGCs; different colors indicate different CNGC groups; (**b**) gene structure representation of *ZjCNGCs*. The rectangles represent exons, and the lines represent introns; (**c**) representation of the protein domains present in 18 ZjCNGCs. Different colors represent different conserved domains.

Individual phylogenetic tree analysis of ZjCNGC revealed that, with the exception of ZjCNGC14, all members of the family were present in the previous evolutionary branch (Figure 1 and Figure 2a). Gene structure analysis of the family members in Group I revealed that the numbers of exons and introns in *ZjCNGC3* are seven and six, respectively; in *ZjCNGC9*, six and five, respectively; and in *ZjCNGC16*, seven and six, respectively (Figure 2b). In the members of Group II, the numbers of exons and introns in *ZjCNGC18* are one and zero, respectively; in *ZjCNGC13*, three and two, respectively, and in *ZjCNGC6*, seven and six, respectively. Among the members of Group III, *ZjCNGC8* has five exons and four introns, *ZjCNGC17* has five exons and four introns, *ZjCNGC15* has seven exons and six introns, *ZjCNGC1* has six exons and five introns, *ZjCNGC12* has six exons and five introns, *ZjCNGC10* has seven exons and six introns, and *ZjCNGC11* has one exon and no introns. Among the Group IVa members, the numbers of exons and introns in *ZjCNGC2* are twelve and eleven, respectively, and the numbers of exons and introns in *ZjCNGC14* are seven and six, respectively. In Group IVb, *ZjCNGC5* has seven exons and six introns, *ZjCNGC4* has seven exons and six introns, and *ZjCNGC7* has three exons and two introns. *ZjCNGC2* was found to have the greatest numbers of exons and introns, whereas *ZjCNGC11* and *ZjCNGC18* had the lowest numbers of exons and introns. Thus, genes belonging to the same groups have different gene structures.

The protein domain analysis results revealed that Group I members contain ion transport, transmembrane, and cNMP-binding domains and that ZjCNGC9 additionally has an IQ domain (Figure 2c). All the members of Group II contain ion transport, transmembrane, and cNMP-binding domains, and ZjCNGC18 has an additional IQ domain within the transmembrane domain. Most members of Group III contain ion transport, transmembrane, cNMP-binding, and IQ domains; however, ZjCNGC11 has only cNMP-binding and IQ domains without the ion transport and transmembrane domains. The transmembrane domain of ZjCNGC12 has a partial cNMP-binding domain. The members of Group IVa contain ion transport, transmembrane, and cNMP-binding domains, and ZjCNGC14 contains an additional IQ domain. The structural domains of the ZjCNGCs in Group IVb are very similar to those of the ZjCNGCs in Group IVa and include ion transport, transmembrane, cNMP-binding, and IQ domains.

### 2.4. Chromosome Location and Collinearity Analysis

**Figure 3:**
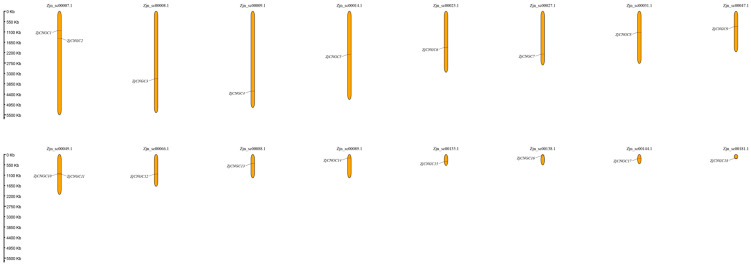
Distribution of *ZjCNGC* genes on *Zoysia japonica* chromosomes at the scaffold level. The chromosome numbers are indicated at the top of each chromosome. The scale on the left is in kilobytes (Kb).

Because complete chromosome information for the genome of *Zoysia japonica* is unavailable, TBtools software (v1.1041) was used to visualize the chromosome positions of the 18 *ZjCNGC* genes at the scaffold level [50]. Chromosome localization analysis revealed that *ZjCNGC* genes are found on all 16 scaffold-level chromosomes of *Zoysia japonica* (Zjn_stc00007.1, 8.1, 9.1, 14.1, 23.1, 27.1, 31.1, 47.1, 49.1, 66.1, 88.1, 89.1, 135.1, 138.1, 144.1, and 181.1) (Figure 3). Zjn_stc00007.1 and Zjn_stc000049.1 each contain two *CNGC* genes, namely *ZjCNGC1* and *ZjCNGC2* and *ZjCNGC10* and *ZjCNGC11*, respectively; these are the scaffold chromosomes with the greatest *CNGC* distribution. Each of the other fourteen chromosomes contains only one *ZjCNGC* gene.

**Figure 4:**
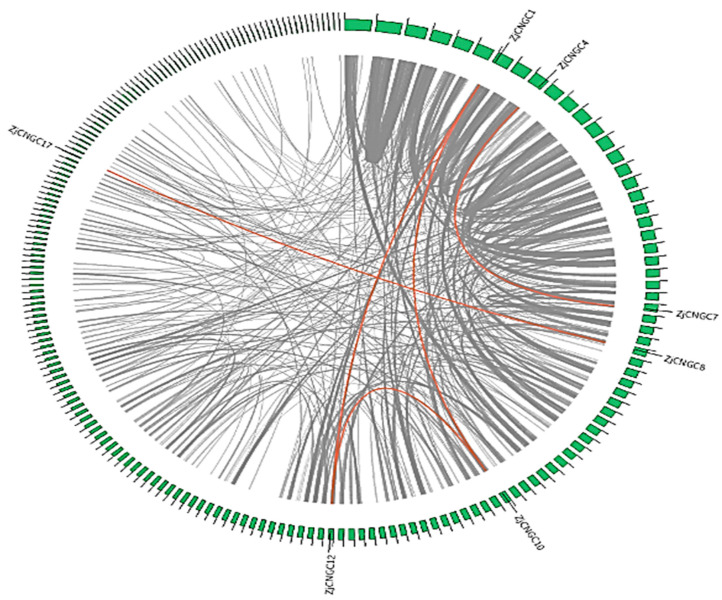
Collinear regions of the *Zoysia japonica CNGC* genes. The grey lines represent all the collinear blocks in the *Zoysia japonica* genome, and the red lines represent *CNGC* gene pairs that have been subjected to segmental duplication.

Intraspecific collinearity analysis was performed on the genome of *Zoysia japonica* using the Multicollinearity Scanning Tool Kit (MCScan X). The results revealed that many collinear gene pairs are present in the genome of *Zoysia japonica* and that five of these collinear gene pairs encode *ZjCNGCs* (Figure 4). The collinear *ZjCNGC* gene pairs are *ZjCNGC1-ZjCNGC10*, *ZjCNGC1-ZjCNGC12*, *ZjCNGC10-ZjCNGC12*, *ZjCNGC8-ZjCNGC17*, and *ZjCNGC4-ZjCNGC7*. *ZjCNGC1*, *8*, *10*, *12*, and *17* belong to Group III, whereas *ZjCNGC4* and *ZjCNGC7* belong to Group IVb (Figure 1). The intraspecific collinearity of *ZjCNGC* gene pairs occurred mainly among the members of Groups III and IVb. The five gene pairs are segmental rather than tandem duplications.

**Figure 5:**
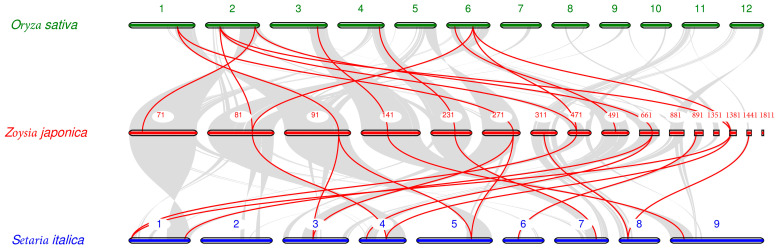
Synteny analysis of *CNGC* genes between *Zoysia japonica* and *Oryza sativa* or *Setaria italica*. The grey lines in the background indicate the collinear blocks within *Zoysia japonica* and other plant genomes; the red lines indicate the syntenic *CNGC* gene pairs.

In the synteny analysis of the genomes of *Zoysia japonica*, rice, and foxtail millet (Figure 5), many synteny gene pairs were found among the three genomes. Thirteen collinear gene pairs between *ZjCNGCs* in *Zoysia japonica* and *OsCNGCs* in rice were found. These include *ZjCNGC1-OsCNGC10*, *ZjCNGC3-OsCNGC1*, *ZjCNGC3-OsCNGC2*, *ZjCNGC4-OsCNGC15*, *ZjCNGC5-OsCNGC14*, *ZjCNGC6-OsCNGC6*, *ZjCNGC7-OsCNGC15*, *ZjCNGC9-OsCNGC1*, *ZjCNGC9-OsCNGC2*, *ZjCNGC10-OsCNGC11*, *ZjCNGC12-OsCNGC10*, *ZjCNGC16-OsCNGC1*, and *ZjCNGC16-OsCNGC2*. Six of the *ZjCNGCs* in these gene pairs are in group I, 1 is in group II, 3 are in group III, and 3 are in group IV. The group III genes of *Zoysia japonica* are *ZjCNGC1*, *ZjCNGC10*, and *ZjCNGC12*. Fifteen synteny gene pairs between *ZjCNGCs* in *Zoysia japonica* and *SiCNGCs* in foxtail millet were found. They are *ZjCNGC3-SiCNGC10*, *ZjCNGC4-SiCNGC7*, *ZjCNGC4-SiCNGC11*, *ZjCNGC5-SiCNGC15*, *ZjCNGC6-SiCNGC13*, *ZjCNGC7-SiCNGC7*, *ZjCNGC7-SiCNGC11*, *ZjCNGC8-SiCNGC14*, *ZjCNGC9-SiCNGC1*, *ZjCNGC12-SiCNGC5*, *ZjCNGC12-SiCNGC9*, *ZjCNGC14-SiCNGC12*, *ZjCNGC16-SiCNGC1*, *ZjCNGC16-SiCNGC10*, and *ZjCNGC17-SiCNGC14*. Of the *ZjCNGC* genes in these pairs, four are in Group I, 1 is in group II, five are in group III, and five are in group IV. The genes in group III of *Zoysia japonica* are *ZjCNGC8*, *ZjCNGC12*, *ZjCNGC14*, and *ZjCNGC17*. The above results collectively indicate that (1) the *CNGCs* of *Zoysia japonica*, rice, and foxtail millet are highly homologous and (2) the expansion of the *ZjCNGC* genes in Group III is partially due to their own evolution (as indicated by the fact that many members of the group III family, such as *ZjCNGC11* and *ZjCNGC15*, have not been found to be collinear in rice and foxtail millet).

### 2.5. Analysis of Cis-Regulatory Elements in the Promoter Region of ZjCNGC Family Genes

**Figure 6:**
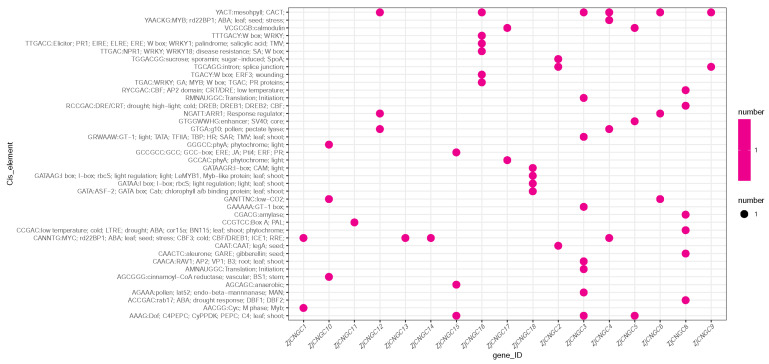
Cis-element statistics of *CNGC* gene family members of *Zoysia japonica*. The horizontal axis is the *CNGC* member ID, and the vertical axis is the cis-element name. The interaction point of the horizontal and vertical coordinates represents the cis-element that interacts with the corresponding *ZjCNGC*.

Promoters contain multiple cis-regulatory elements (CREs) that regulate plant development and physiological processes by modulating gene expression [51]. To identify CREs in the promoter sequence of the *ZjCNGC* genes, the TBtools program was used to extract 2 kb of the sequence upstream of each *CNGC* member as the promoter region sequence. The PlantCare program was subsequently used to predict the cis-regulatory elements located in these promoter regions. The results revealed that CREs are present in the promoter regions of most *ZjCNGCs*, with the exception of *ZjCNGC7*, which has no CREs. The *CNGC* family member whose promoter region contains the most CREs is *ZjCNGC3*, for which there are eight predicted CREs, followed by *ZjCNGC8* and *16*, for each of which there are six predicted CREs. The *CNGC* family members whose promoter regions contain the fewest CREs are *ZjCNGC11*, *13*, and *14*; the promoter regions of each of these genes contain 1 predicted CRE (Figure 6). The predicted CREs include the following stress categories: stress (YAACKG, CANNTGC), salt (GAAAAA), pathogen (TGAC, GAAAAA, and GCCGCC), disease resistance (TTGAC), injury (TGACY), low temperature (RYCGAC), cold (RCCGAC and CANNTGC), drought (RCCGAC, CCGAC, and ACCGAC), low CO2 (GANTCC), and anaerobic (AGCGC). The results indicate that *ZjCNGC* gene expression is regulated by various stresses, including salt stress, at the transcriptional level, suggesting that *ZjCNGCs* play a role in plant stress resistance. In the CRE prediction results, only one directly salt-related CRE was found; that was GAAAAA in the *ZjCNGC3* promoter region. *CNGC* is also an important calcium ion transport channel that plays a crucial role in maintaining intracellular calcium ion levels, and *CaM* (calmodulin) is an important binding protein of intracellular calcium ions. By binding to calcium ions, *CaM* is activated and participates in regulating downstream signaling pathways that help cells cope with changes in the external environment [25]. Therefore, *CaM*-related CREs are important sites for regulating the expression and function of *CNGC* genes. Cis-element prediction revealed that in the first 2 kb of the promoter regions of the genes encoding *ZjCNGC3* and *ZjCNGC18*, CAM-related CREs were found; these were GAAAAA, GATAAGR and GATA.

### 2.6. Expression of ZjCNGC Family Genes in Plant Tissues

**Figure 7:**
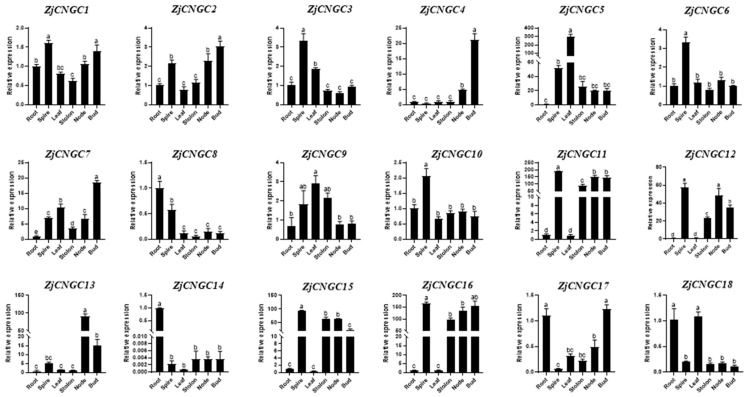
Expression patterns of genes belonging to the *ZjCNGC* gene family in various tissues of *Zoysia japonica*. Error bars indicate the standard deviation (SD) for three biological replicates based on qRT–PCR. Significant differences among treatments were indicated by different letters (one-way ANOVA analysis, *p* < 0.05).

The expression of 18 *ZjCNGC* genes in various tissues of *Zoysia japonica* (roots, spires, leaves, stolons, stem nodes, and buds) was measured via qRT–PCR [50]. The qPCR results revealed that *ZjCNGCs* are expressed in all of these tissues, but their expression levels in the different tissues vary (Figure 7). In roots, *ZjCNGC 8*, *14*, *17*, and *18* were the most highly expressed *ZjCNGC* genes. The spire showed highest expression of *ZjCNGC1*, *3*, *6*, *10*, *11*, *12*, *15*, and *16*, while *ZjCNGC5*, *9*, and *17* were the *ZjCNGC* genes that were most highly expressed in leaves. In stem nodes, *ZjCNGC12* and *ZjCNGC13* were the most highly expressed, whereas in buds, *ZjCNGC1*, *2*, *4*, *7*, and *18* were the most highly expressed. Among them, *ZjCNGC1*, *12*, *17*, and *18* presented the highest expression in two different tissues. *ZjCNGC6* and *10* were highly expressed only in the spire, with little difference in expression levels in other tissues. *ZjCNGC14* was most highly expressed in the roots. The expression patterns of *ZjCNGC* in different tissues revealed that eight of the *ZjCNGCs* presented the highest expression levels in the spike and that the majority of the *ZjCNGC* genes were expressed at low levels in the stolon.

### 2.7. Expression of ZjCNGC Family Genes in Response to Salt Stress

**Figure 8:**
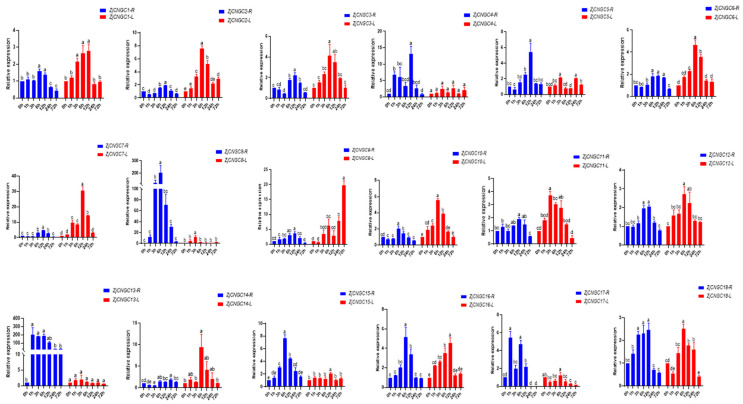
Relative expression levels of *ZjCNGC* genes in roots and leaves after exposure of plants to salt stress. Significant differences among treatments were indicated by different letters (one-way ANOVA analysis, *p* < 0.05). The letter R indicated the roots and the letter L indicated the leaves.

The relative expression levels of the *ZjCNGC* genes in roots and leaves at 0 h, 1 h, 3 h, 6 h, 12 h, 24 h, and 72 h after the initiation of exposure to salt stress were measured via qRT–PCR (Figure 8). The results revealed that expression of these 18 *ZjCNGC* genes in the roots first increased but then decreased with increasing salt treatment time, indicating that *ZjCNGC* may be involved in regulation of the salt response. In roots, *ZjCNGC13* and *ZjCNGC17* reached their maximum expression levels 1 h after the initiation of salt treatment (Figure 8), whereas *ZjCNGC13* reached its maximum expression level 3 h after the initiation of salt treatment. The expression of *ZjCNGC1*, *ZjCNGC6*, *ZjCNGC8*, *ZjCNGC10*, *ZjCNGC12*, *ZjCNGC15*, and *ZjCNGC16* in the roots peaked 6 h after salt treatment. At 12 h after the initiation of salt stress, the expression levels of *ZjCNGC2*, *ZjCNGC3*, *ZjCNGC4*, *ZjCNGC5*, *ZjCNGC7*, *ZjCNGC9* and *ZjCNGC11* in the roots peaked. The expression of *ZjCNGC14* in roots reached its maximum 24 h after the initiation of salt stress.

After the initiation of salt stress, the expression levels of almost all *ZjCNGCs* in leaves tended to first increase but then decrease, except those of *ZjCNGC4* and *ZjCNGC13*, whose expression did not change significantly (Figure 8). The expression levels of four *ZjCNGC genes* (*ZjCNGC1*, *ZjCNGC5*, *ZjCNGC8*, and *ZjCNGC11*) in leaves reached their maximum values 3 h after salt treatment began, whereas the expression levels of eight *ZjCNGC* genes (*ZjCNGC2*, *ZjCNGC3*, *ZjCNGC6*, *ZjCNGC10*, *ZjCNGC12*, *ZjCNGC14*, *ZjCNGC17*, and *ZjCNGC18*) in leaves reached their maximum values 6 h after salt treatment. The expression levels of *ZjCNGC4*, *7*, *9*, *15*, and *16* in the leaves peaked after 12 h of salt stress. Notably, the expression level of *ZjCNGC9* in leaves reached a maximum after 72 h of salt stress.

### 2.8. Predicted Interactions between ZjCNGCs and Other Proteins

**Figure 9:**
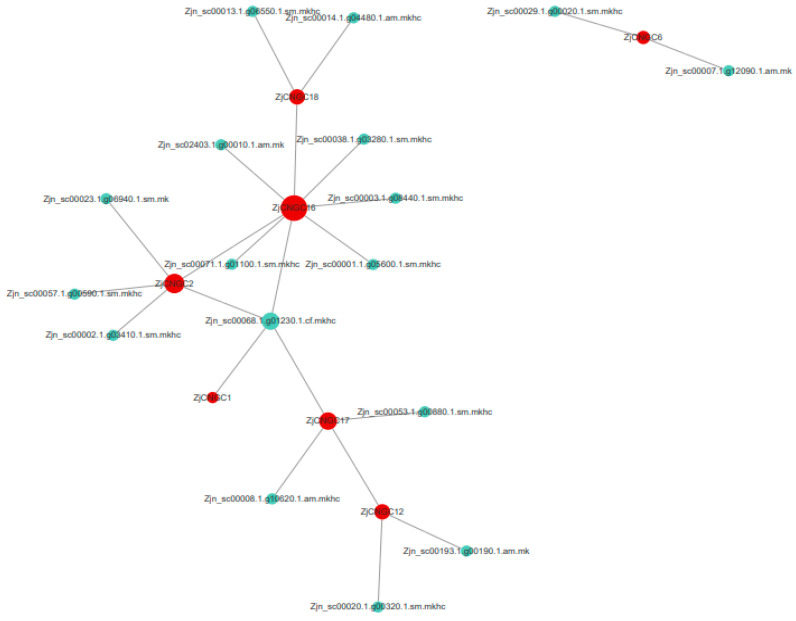
PPI networks involving ZjCNGC2, ZjCNGC6, ZjCNGC12, ZjCNGC16, ZjCNGC17 and ZjCNGC18. The red circles represent ZjCNGC proteins, and the light green circles represent predicted interacting proteins. Larger circles representing the proteins indicate stronger functional relationships.

To predict which protein interact with ZjCNGCs, protein–protein interaction (PPI) networks were constructed on the basis of homologous Arabidopsis genes. As shown in Figure 9, seven ZjCNGCs (ZjCNGC1, ZjCNGC2, ZjCNGC6, ZjCNGC12, ZjCNGC16, ZjCNGC17, ZjCNGC18) were predicted to interact with other proteins. No predicted interacting proteins were found for the other 11 ZjCNGCs. ZjCNGC1 has one predicted interacting protein [polyubiquitin 3 (UBQ3)], ZjCNGC2 has five predicted interacting proteins [NDR1/HIN1-like 2 (NHL2), ZjCNGC16, polyubiquitin 3 (UBQ3), calmodulin 5, and cytokine/deoxycytidine deaminase family protein], and ZjCNGC6 has two predicted interacting proteins (prenylcysteine methylesterase and long-chain fatty alcohol dehydrogenase family protein). ZjCNGC12 has three predicted interacting proteins (H^+^-ATPase 2, ZjCNGC17, and somatic embryogenesis receptor-like kinase 1), ZjCNGC16 has eight predicted interacting proteins (Ca^2+^-activated outwardly rectifying K^+^ channel 6, autoinhibited Ca^2+^-ATPase 1, polyol/metabolite transporter 5, lysine histidine transporter 1, polyubiquitin 3, ZjCNGC2, ZjCNGC18, and an unknown protein), ZjCNGC17 has four predicted interacting proteins (polyubiquitin, seven transmembrane MLO family protein, calmodulin-domain protein kinase 7, and ZjCNGC12), and ZjCNGC18 has three predicted interacting proteins (calcium-dependent protein kinase 1, outwardly rectifying potassium channel protein, and ZjCNGC16). According to the results, the main proteins that interact with ZjCNGC are ZjCNGC, calcium-dependent protein kinase, H^+^-ATPase, outwardly rectifying potassium channel protein, and polyubiquitin 3.

## 3. Discussion

### 3.1. Using Scaffold-Level Genomes in Gene Family Analysis Requires Special Caution

Published genomic information on *Zoysia japonica* is available only at the scaffold level, and there may be problems such as errors related to chromosome splicing and annotation errors, resulting in incomplete sequences and errors in the identification of genes within the *Zoysia japonica* genome [51]. As a result, the genes obtained from the *Zoysia japonica* genome may contain sequence errors and incomplete sequence information, seriously interfering with subsequent research on the genes of *Zoysia japonica*. By searching for CNGC proteins in the genome of *Zoysia japonica* using the CNGC conserved domain CNBD and comparing their sequences with third-generation full-length transcriptome *CNGC* sequences, it was found that only four *ZjCNGC* genes in the *Zoysia japonica* genome are consistent with the third-generation full-length transcriptome sequence. The other 14 *ZjCNGCs* were reannotated through transcriptomics and by referencing the Arabidopsis and rice *CNGC* genes. Using human correction methods, 18 highly accurate *ZjCNGC* gene sequences of *Zoysia japonica* were ultimately obtained (Table 1; Figure 1). The results we obtained indicate that the use of scaffold-level genomes in gene family analysis requires special caution. Moreover, the use of a combination of genomic information, third-generation full-length transcriptomes, and human-corrected genome methods in this study represents a new approach to obtaining high-precision sequence information for species whose genomic information has problems such as genome splicing and incomplete gene information.

### 3.2. ZjCNGCs Participate in the Salt Stress Response of Zoysia japonica

CNGC proteins are important cation transport channels in plants; they participate in regulating the plant’s responses to various biotic and abiotic stresses such as drought, cold, high temperature, and salt stress by forming homologous or heterologous tetrameric ion channels [26,33,40,41,52]. By predicting the cis-acting elements in the promoter regions of *ZjCNGC* genes, multiple stress response elements, including those involved in salt stress, were identified (Figure 6). The quantitative analysis revealed that the transcript abundances of almost all *ZjCNGCs* were upregulated after salt stress, but with distinct response times (Figure 8). The expression levels of *ZjCNGC8* and *ZjCNGC13* changed significantly after salt treatment. The expression level of *ZjCNGC8* reached a maximum after 6 h of salt treatment; at that point, its relative expression was 201.61 times greater than the level of expression in the absence of salt treatment. The expression level of *ZjCNGC13* reached a maximum after 1 h of salt treatment; at that point, its relative expression was 202.60 times greater than the level in the absence of salt treatment. These results collectively indicate that *ZjCNGCs* respond to salt stress through changes in their transcription levels. In combination with the results regarding the expression of specific members of the *ZjCNGC* family in different tissues (Figure 7), it is speculated that *ZjCNGCs* in *Zoysia japonica* achieve different divisions of labor through differences in the tissues in which they are expressed and differences in their salt stress response patterns, thereby enabling the plant to undergo a stress response.

The functional form of CNGC is a heterotetramer [25]. The PPI protein interaction network diagram indicates that ZjCNGC16 may interact with ZjCNGC2 and 18, and it is speculated that ZjCNGC16 may interact with ZjCNGC2 and 18 and then form a CNGC heterotetramer to exert its effect. Calcium-dependent protein kinase, H^+^-ATPase, outwardly rectifying potassium channel protein, and polyubiquitin 3 are also predicted to interact with CNGCs (Figure 9). CaM and CPK are important proteins that are regulated by Ca^2+^ in cells. Ca^2+^ influx into the cytoplasm mediated by CNGCs activates downstream signal amplification reactions of the CaM and CPK protein cascades in response to external stimuli. On the other hand, the activated CaM protein binds to the CaMBD domain of CNGC [25], closing the CNGC protein channel to prevent excessive Ca^2+^ influx and turning off signal amplification [17,19,25,53]. There are reports that AtCNGC17 interacts with AHAs to form a functional cation transport unit that can be activated by PSKR1/BAK1 and other possible BAK1/RLK complexes to regulate plant growth [39]. Here, the interaction between ZjCNGC12 and AHA2 was also predicted. UBQ3 (polyubiquitin protein 3) interacts with multiple ZjCNGC proteins, including ZjCNGC1, 2, 16, and 17. UBQ3 participates in protein degradation by clearing unwanted, damaged, and other useless proteins from cells through the ubiquitin proteasome pathway [54,55]. UBQ3 may degrade excessive or damaged ZjCNGC proteins in cells through the ubiquitin proteasome pathway, effectively enhancing the response of *Zoysia japonica* to salt stress. These results collectively indicate that ZjCNGCs can also respond to salt stress through functional regulation at the protein level.

It has been reported that *CNGC* genes are involved in the regulation of plant salt tolerance. For example, *AtCNGC19* and *AtCNGC20* respond to cellular salt damage by transporting Ca^2+^ and Na^+^ from vacuoles to the cytoplasm. *AtCNGC3* may increase the salt tolerance of *Arabidopsis thaliana* by transporting Na^+^ and K^+^, whereas *AtCNGC10* plays a negative regulatory role in the salt tolerance of *Arabidopsis thaliana* [25]. VIGS experiments in cotton confirmed that silencing of *GhCNGC32* and *GhCNGC35* can reduce the salt tolerance of cotton [56]. In *Amaranthus hypochondriacus* L., *AhCNGC5* expression was upregulated and *AhCNGC17* expression was downregulated in response to salt stress [57]. The transcriptional and protein regulatory responses of *ZjCNGC* genes to salt stress reported in this study suggest that these genes can also be manipulated as salt tolerance genes to ultimately improve salt tolerance in *Zoysia japonica*. *ZjCNGC8* and *ZjCNGC13* are considered the preferred genes because they show the strongest response to salt stress in roots.

### 3.3. Gene Expansion in Group III of ZjCNGC May Play an Important Role in the Salt Tolerance of Zoysia japonica

The members of the *CNGC* family present in *Zoysia japonica* can be divided into four groups, namely, I, II, III, and IV. The protein phylogenetic tree revealed that *Arabidopsis thaliana* has five Group III members (AtCNGC14, 15, 16, 17, and 18) members, rice has five (OsCNGC7, 8, 9, 10, and 11), foxtail millet has six (SiCNGC3, 5, 6, 9, 12, and 14), and *Zoysia japonica* has eight (ZjCNGC1, 8, 10, 11, 12, 14, 15, and 17) (Figure 1 and Figure 2). Comparison with the protein evolutionary trees of Arabidopsis, rice, and foxtail millet shows that the number of CNGC Group III gene family members is greatest in *Zoysia japonica*; this may represent a phenomenon of gene expansion. The expansion of Group III genes in *Zoysia japonica* is further supported by the findings that only five CNGC Group III members are present in maize and sugarcane [58] and only six are present in Chinese cabbage [41]. The expression of the Group III ZjCNGC genes in *Zoysia japonica* is upregulated under salt stress conditions (Figure 8), and interacting proteins that regulate its protein channel activity are predicted (Figure 9), suggesting that Group III, which has undergone gene expansion, plays an important role in salt tolerance in *Zoysia japonica*.

The intraspecies collinearity results indicate that segmental gene duplication events are important causes of the expansion of gene family members in Group III (Figure 4). The results indicate that only some of the *CNGC* family members in rice and millet are collinear with Group III *CNGC* genes in *Zoysia japonica* (Figure 5), suggesting that some of the *Zoysia japonica* Group III genes were obtained through special evolutionary events. The Group III member *ZjCNGC11*, which does not exhibit interspecies collinearity, serves as an example. Unlike other ZjCNGC proteins, which contain 2–7 transmembrane domains, ZjCNGC11 does not contain transmembrane domains, only contain important functional regulatory domains such as IQ and cNMP-binding domains (Figure 2). Members of the CNGC family are important transmembrane proteins, yet *Zoysia japonica* possesses a gene encoding a transmembrane domain-free member of the CNGC family, ZjCNGC11, which was found to be located in Group III during gene expansion. This transmembrane domain-free form of CNGC may be a new evolutionary form that diversifies the regulation of ZjCNGC family genes in response to external stresses such as salt stress. In the presence of intracellular free CNGCs, the functional forms of CNGC tetramers may have become more diverse to allow the plant to cope with more complex changes in the external environment.

There have been reports of an association between Arabidopsis *AtCNGC17*, a member of Group III, and salt tolerance in Arabidopsis [19,25]. Because gene expansion has occurred among the Group III family members of *Zoysia japonica*, their gene expression is sharply upregulated under salt stress (Figure 8). This group exhibits only partial collinearity with Group III genes in other closely related species, and ZjCNGC11 has a unique protein structure, collectively suggesting that gene expansion in Group III of *Zoysia japonica* may be a unique strategy employed by plants for salt tolerance.

## 4. Materials and Methods

### 4.1. Identification of CNGC Genes in Zoysia japonica

Potential CNGC proteins were retrieved from the hidden Markov model profiles corresponding to the ion transport (PF00520.30) or cNMP-binding (PF00027.28) domain from the Pfam protein family database (http://pfam.xfam.org/search) via HMMER (http://HMMER.org/download.html) [59]. A BLASTP algorithm (e value < 1 × 10^−5^) was subsequently applied to identify the CNGC proteins in the UniProt database (https://www.uniprot.org/). The genomic data of *Zoysia japonica* (Nagirizaki r1.1), *Setaria italica* (Setaria italica v2.2), Oryza sativa (Oryza sativa v7.0) and *Arabidopsis thaliana* (Arabidopsis thaliana Araport11) were downloaded from the Zoysia Genome Database (https://zoysia.kazusa.or.jp/index.html) and Phytozome 13 (https://phytozome-next.jgi.doe.gov/). The raw PacBio sequencing data of three generations of the full-length transcriptome database have been deposited at the National Center for Biotechnology Information (NCBI) under Bioproject ID PRJNA1132803. The nonredundant transcripts (identity > 0.99) were obtained via the program CD-HIT v4.8.1 (http://weizhongli-lab.org/cd-hit/). Improved genome annotations were derived using MAKER. The SMART (Simple Modular Architecture Research Tool, http://smart.embl-heidelberg.de/) database was used to verify the presence of ion transport, transmembrane, cNMP-binding and IQ domains [60]. Finally, the physiochemical properties of the ZjCNGC proteins, including their amino acid sequences, molecular weights (MWs), isoelectric points (pIs), and grand average hydropathicity (GRAVY), were examined using the ProParam tool at the ExPASy website (https://web.expasy.org/protparam/) [61].

### 4.2. Phylogenetic Analysis of CNGC Genes in Arabidopsis, Rice, Foxtail Millet, and Zoysia japonica

The CNGC protein sequences of *Zoysia japonica*, rice, Arabidopsis, and millet were compared with the default parameter (pairwise deletion, 1000 bootstrap) in the multisequence alignment tool Muscle software (v3.8.31). A maximum-likelihood (ML) phylogenetic tree was constructed using MEGA 7.0 software, and the parameter used was the p-distance [62]. The phylogenetic tree was visualized using the itols program (http://itol.embl.de) [63].

### 4.3. Analysis of Gene Structure and Conserved Motifs in ZjCNGCs

The exon–intron structure of the *CNGC* gene was analyzed via online tools (http://gsds.cbi.pku.edu.cn/). The PfamScan program (http://pfam.xfam.org/) was used for online motif analysis, and the WebLogo online platform (http://weblogo.berkeley.edu) was used for sequence marker analysis of the conserved domain.

### 4.4. Chromosomal Location and Collinearity Analysis of ZjCNGC Genes

The position of each *CNGC* gene on the 16 *Zoysia japonica* chromosomes was determined from the *Zoysia japonica* genome database, and a genetic linkage map was constructed using TBtools [64].

MCScanX software (version 2.0)was used to analyze and identify the protein and GFF files of the *Zoysia japonica*, rice, and Arabidopsis genomes, and Circos v0.55 was used for visual analysis [65].

### 4.5. Promoter Analysis of ZjCNGC Genes

To determine the number of cis-elements in the *ZjCNGC* gene promoter, the upstream sequence (2 kb) of the start codon of each gene was extracted from the *Zoysia japonica* genome sequence using bedtools (v2.26.0) software. The cis-elements of these gene promoter regions were analyzed using PlantCARE software (http://bioinformatics.psb.ugent.be/webtools/plantcare/html/). Visualization was achieved using the ggplot2 package.

### 4.6. Expression of ZjCNGC Genes

Stolons of the *Zoysia japonica* (accession: Z011) were collected from field plots in the turfgrass nursery in Nanjing Botanical Garden Mem. Sun Yat-Sen, China (32.055° N, 118.834° E). Stolons with their top four nodes were cultured in water for 7 days to allow root emergence. Uniform seedlings were then subjected to treatment with 1/2 Hoagland’s solution under hydroponic conditions in a greenhouse. The light/dark cycle was 14 h/10 h, and the relative humidity was 70%. After 35 d of treatment, the roots, young leaves, leaves, stolons, stem nodes, and buds of *Zoysia japonica* were harvested separately and used to analyze the tissue-specific expression of *ZjCNGCs*. The plants that had been pretreated for 35 d were then subjected to treatment with 1/2 Hoagland’s solution supplemented with 350 mM NaCl by changing the nutrient solution. The roots and leaves were harvested at following salt-treated time point: 0 h, 1 h, 3 h, 6 h, 12 h, 24 h, and 72 h, and used in salt–response analysis of *ZjCNGCs*. The samples were ground into powder in liquid nitrogen. Total RNA was isolated using RNAiso (Takara, Kusatsu, Japan) and treated with RNase-free DNase I (Takara, Japan) for 15 min to eliminate potential contaminating DNA. The RNA quality was checked by the agarose gel electrophoresis and A260/A280 measurement. One microgram of RNA was reverse-transcribed into cDNA using a Prime Script RT kit (Takara, Japan), and gene expression analysis was performed via real-time quantitative PCR (qPCR). qPCR was performed using the Step One Real-Time PCR System (Applied Biosystems, Waltham, MA, USA). The PCR protocol was as follows: 95 °C for 30 s followed by 40 cycles of 95 °C for 5 s and 60 °C for 30 s. The raw qRT-PCR results were obtained by the BioRad CFX Maestro software (version 2.3). Relative gene expression was calculated using the 2^−ΔΔCt^ method [66]. Each treatment included three biological replicates. The software Primer (version 5.0) was used for primer design and primers information was listed in Supplementary Table S1. The reference gene used was ZjActin [67]. The significance analysis was conducted using a one-way analysis of variance (ANOVA) in SPSS version 26.0, with a significance level set at *p* < 0.05.

### 4.7. Analysis of Protein Interactions

The BLASTP program (version 2.0) (threshold < 1 × 10^−5^) was used to compare all protein sequences with the String interaction database, and proteins with interaction scores greater than 200 were extracted. The network diagram was drawn using the Cytoscape program (version 3.3).

## 5. Conclusions

With the help of the genome, third-generation full-length transcriptome, and artificial calibration of *Zoysia japonica*, a total of 18 *CNGCs* were identified in this study. This method provides a methodological reference for gene family analysis in species with poorly assembled genomes. Compared with the *CNGC* group III of other species, the *CNGC* group III of *Zoysia japonica* has undergone gene expansion, primarily through gene segmental duplication. The results of interspecific collinearity analysis suggest that the expansion of group III *ZjCNGCs* may be an independent evolutionary event occurring in *Zoysia japonica*. The expression of individual *ZjCNGCs* in specific tissues differed, but most members of the *ZjCNGC* gene family showed upregulated expression in the roots and leaves in response to salt stress. These genes may play a role in the salt tolerance of *Zoysia japonica*, and manipulating these genes is expected to improve plant salt tolerance. The gene expansion phenomenon that occurred in group III may be a unique strategy by which the halophilic plant *Zoysia japonica* has adapted to salt stress environments.

## Figures and Tables

**Table 1:** Characteristics of the *CNGC* gene in *Zoysia japonica*.

Gene_ID	Nagirizaki Re Annotated Genome	AA (aa)	MW (kDa)	pI	II	Gravy	Scaffold Location	Protein Domain	TMHs	Subcellular Localization
*ZjCNGC1*	Zjn_sc00007.1_PB.1.1 gene = PB.1	692	79.10	9.53	43.30	−0.09	Zjn_sc00007.1: 1025077–1029126	Ion_trans + cNMP	5	Chloroplast
*ZjCNGC2*	Zjn_sc00007.1_00002.1 gene = Zjn_sc00007.1_00002	755	86.91	8.83	53.94	−0.14	Zjn_sc00007.1: 1450038–1455783	Ion_trans + cNMP	5	Chloroplast
*ZjCNGC3*	Zjn_sc00008.1_PB.3.1 gene = PB.3	702	81.17	9.98	52.15	−0.10	Zjn_sc00008.1: 3595513–3601503	Ion_trans + cNMP	5	Plasma membrane
*ZjCNGC4*	Zjn_sc00009.1_PB.4.1 gene = PB.4	675	75.73	10.21	46.97	−0.09	Zjn_sc00009.1: 4230624–4233998	Ion_trans + cNMP	7	Chloroplast
*ZjCNGC5*	Zjn_sc00014.1_PB.5.1 gene = PB.5	719	80.71	9.27	56.34	0.09	Zjn_sc00014.1: 2287261–2292041	Ion_trans + cNMP	6	Chloroplast
*ZjCNGC6*	Zjn_sc00023.1_00001.1 gene = Zjn_sc00023.1_00001	720	82.49	8.87	47.02	−0.15	Zjn_sc00023.1: 1913507–1917920	Ion_trans + cNMP	7	Plasma membrane
*ZjCNGC7*	Zjn_sc00027.1_PB.7.1 gene = PB.7	703	79.08	10.00	52.85	−0.17	Zjn_sc00027.1: 2283235–2286748	Ion_trans + cNMP	6	Chloroplast
*ZjCNGC8*	Zjn_sc00031.1.g02390.1.sm.mk	637	72.31	8.70	38.09	−0.20	Zjn_sc00031.1: 1120713–1123586	Ion_trans + cNMP	4	Chloroplast
*ZjCNGC9*	Zjn_sc00047.1_00001.1 gene = Zjn_sc00047.1_00001	637	72.72	8.13	48.89	0.05	Zjn_sc00047.1: 821511–824430	Ion_trans + cNMP	5	Plasma membrane
*ZjCNGC10*	Zjn_sc00049.1_PB.8.1 gene = PB.8	686	79.38	9.45	43.53	−0.09	Zjn_sc00049.1: 1033362–1037607	Ion_trans + cNMP	5	Plasma membrane
*ZjCNGC11*	Zjn_sc00049.1.g02530.1.sm.mk	179	20.52	9.55	38.34	−0.26	Zjn_sc00049.1: 1044885–1045421	partial cNMP	0	Chloroplast
*ZjCNGC12*	Zjn_sc00066.1_PB.9.1 gene = PB.9	692	79.29	9.25	50.61	−0.15	Zjn_sc00066.1: 1047067–1050019	Ion_trans + cNMP	6	Extracellular
*ZjCNGC13*	Zjn_sc00088.1_00001.1 gene = Zjn_sc00088.1_00001	766	86.95	9.46	51.70	−0.14	Zjn_sc00088.1: 489510–492207	Ion_trans + cNMP	3	Chloroplast
*ZjCNGC14*	Zjn_sc00089.1_00001.1 gene = Zjn_sc00089.1_00001	628	71.53	8.55	41.12	−0.05	Zjn_sc00089.1: 181512–185301	Ion_trans + cNMP	5	Plasma membrane
*ZjCNGC15*	Zjn_sc00135.1.g00520.1.am.mkhc	713	81.87	9.34	47.86	−0.20	Zjn_sc00135.1: 380723–385773	Ion_trans + cNMP	4	Nucleus
*ZjCNGC16*	Zjn_sc00138.1_PB.11.1 gene = PB.11	700	80.44	8.67	47.39	−0.09	Zjn_sc00138.1: 75078–80192	Ion_trans + cNMP	5	Plasma membrane
*ZjCNGC17*	Zjn_sc00144.1_00001.1 gene = Zjn_sc00144.1_00001	732	83.20	8.49	42.58	−0.15	Zjn_sc00144.1: 232593–235715	Ion_trans + cNMP	5	Chloroplast
*ZjCNGC18*	Zjn_sc00181.1.g00240.1.sm.mk	713	81.15	9.64	53.73	−0.11	Zjn_sc00181.1: 207414–209552	Ion_trans + cNMP	2	Chloroplast

## Data Availability

The data presented in this study are available on request from the corresponding author.

## Supplementary Materials

The supporting information can be downloaded at: https://www.mdpi.com/article/10.3390/ijms251810114/s1.
