# Supplementary material for: Genome-Wide Identification and Expression Analysis of the Cyclic Nucleotide-Gated Channel Gene Family in Zoysia japonica under Salt Stress
Source: Int J Mol Sci. 2024 Sep 20;25(18):10114. doi: 10.3390/ijms251810114 (PMC11432434; doi:10.3390/ijms251810114)
Supplement: Supplementary file 1 [file ijms-25-10114-s001.zip › ijms-3213478-supplementary.pdf]

Supplementary table 1. Primers used for qPCR.

Name	Primer (5'→3')	Target Sequence Size（bp）
ZjActin-F	CACTGTGCCAATCTATGAAGGA	126
ZjActin-R	GAGGTTGTGAATGAGTAACCCC	
ZjCNGC1-F	GTTCTGACTTAGCCGTTGAT	89
ZjCNGC1-R	AGTGGAATACTTTATAGCTGGT	
ZjCNGC2-F	ATTGCCCTCCTGCTTGTAG	117
ZjCNGC2-R	CCCTAATCTTCTTCGTCCA	
ZjCNGC3-F	TTGATGTCTTCGCCGTTAT	136
ZjCNGC3-R	ATTAGCCGAGGCACATACT	
ZjCNGC4-F	CGACACCGATGCCGACGAG	267
ZjCNGC4-R	AAGCGGACCGAGTGGTAGAT	
ZjCNGC5-F	CGTCGTCCTGCCAATCCCG	207
ZjCNGC5-R	AGACCAAATCCCCACCATA	
ZjCNGC6-F	CATTGCTACCACTTCCACA	109
ZjCNGC6-R	TACTGAATCAAGACAACCCA	
ZjCNGC7-F	GATGAGCAGGACCAGGAGGG	195
ZjCNGC7-R	GAGCAAGAACGCCACCAGC	
ZjCNGC8-F	ATCGTAAAGGCAAGTGGAG	321
ZjCNGC8-R	CAGGGCATCGGAGAATAAC	
ZjCNGC9-F	GGCTTCGTTGCTCCTTCTG	184
ZjCNGC9-R	TCCTTGCCTTCATAACCTCT	
ZjCNGC10-F	GCCTCATTACCTTTACCAC	100
ZjCNGC10-R	GAGCAACGAGAACCAGCAT	
ZjCNGC11-F	CGCTACTACTCGCACCACT	153
ZjCNGC11-R	CCATTCCAACGACGCCAAA	
ZjCNGC12-F	CGACTATGAGGGTGTTTGG	200
ZjCNGC12-R	AGCAAGAACTATGACCACCA	
ZjCNGC13-F	CAGGTCAACATCTGGCACTTT	241
ZjCNGC13-R	GAAGATACCACAACGAACCCA	
ZjCNGC14-F	GCATCGTGCTCACTTGTCT	316
ZjCNGC14-R	GCTCCATCCCAGCCATTTT	
ZjCNGC15-F	GCGTTCACTTGCTGACTTG	370
ZjCNGC15-R	ATTGTATGCTGCCCCTCCC	
ZjCNGC16-F	TAGAGTGTTTGGTCGGGGTGT	244
ZjCNGC16-R	TGTGATTTGGAGGTAAAGTGG	
ZjCNGC17-F	CGTCGATCCGCTCTACTTC	427
ZjCNGC17-R	GACTACTCCGCTTGCCTTT	
ZjCNGC18-F	GCCACCTTCGGTCTCATCC	265
ZjCNGC18-R	AGAGGTGTCGCTTGATGTCG	
